# Supplementary material for: Prediction models for the prediction of unplanned hospital admissions in community-dwelling older adults: A systematic review
Source: PLoS One. 2022 Sep 23;17(9):e0275116. doi: 10.1371/journal.pone.0275116 (PMC9506609; doi:10.1371/journal.pone.0275116)
Supplement: S1 File — (PDF) [file pone.0275116.s002.pdf]

S1 File: Full search strategies

PubMed Session Results (07 Jan 2021)

Search	Query	Items found
#5	**#4 AND (("2013/08/01"[EDAT] : "3000/01/01"[EDAT]) OR ("2013/08/01"[PDAT] : "3000/01/01"[PDAT]))**	5,780
#4	**#1 AND #2 AND #3**	9,680
#3	**(validat*[tiab] OR predict*[ti] OR rule*[tiab]) OR (predict*[tiab] AND (outcome*[tiab] OR risk*[tiab] OR model*[tiab])) OR ((history[tiab] OR variable*[tiab] OR criteria[tiab] OR scor*[tiab] OR characteristic*[tiab] OR finding*[tiab] OR factor*[tiab]) AND (predict*[tiab] OR model*[tiab] OR decision*[tiab] OR identif*[tiab] OR prognos*[tiab])) OR (decision*[tiab] AND (model*[tiab] OR clinical*[tiab] OR "Logistic Models"[Mesh])) OR (prognostic[tiab] AND (history[tiab] OR variable*[tiab] OR criteria[tiab] OR scor*[tiab] OR characteristic*[tiab] OR finding*[tiab] OR factor*[tiab] OR model*[tiab]))**	4,237,645
#2	**"Home Health Nursing"[Mesh] OR "Community Health Nursing"[Mesh] OR "Independent Living"[Mesh] OR "Aged, 80 and over"[Majr] OR "Frail Elderly"[Majr] OR "Frailty"[Majr] OR "community dwell*"[tiab] OR "home dwell*"[tiab] OR "living at home"[tiab] OR "aging in place"[tiab] OR "ageing in place"[tiab] OR ("independent*"[tiab] AND ("living"[tiab] OR "housing"[tiab])) OR "home care*"[tiab] OR "district nurs*"[tiab] OR "visiting nurs*"[tiab] OR "health visitor*"[tiab] OR "community health nurs*"[tiab] OR "elderly"[tiab] OR "eldest"[tiab] OR "late-life*"[tiab] OR "frail"[tiab] OR "frailty"[tiab] OR "old age*"[tiab] OR "oldest old*"[tiab] OR "senior*"[tiab] OR "senium"[tiab] OR "very old*"[tiab] OR "septuagenarian*"[tiab] OR "octagenarian*"[tiab] OR "octogenarian*"[tiab] OR "nonagenarian*"[tiab] OR "centarian*"[tiab] OR "centenarian*"[tiab] OR "supercentenarian*"[tiab] OR "older people"[tiab] OR "older subject*"[tiab] OR "older patient*"[tiab] OR "older age*"[tiab] OR "older adult*"[tiab] OR "older man"[tiab] OR "older men"[tiab] OR "older male*"[tiab] OR "older woman"[tiab] OR**	603,028

Search	Query	Items found
	"older women"[tiab] OR "older female*"[tiab] OR "older population*"[tiab] OR "older person*"[tiab] OR "older individual*"[tiab] OR "aged person*"[tiab]	
#1	"Hospitalization/statistics and numerical data"[Majr:NoExp] OR "Emergency Service, Hospital/statistics and numerical data"[Majr] OR "Patient Admission"[Majr] OR "Patient Readmission"[Mesh] OR "hospital admission*"[tiab] OR "hospital readmission*"[tiab] OR "hospital re-admission*"[tiab] OR "hospital entry*"[tiab] OR "hospital re-ent*"[tiab] OR (hospitali*[tiab] AND predict*[tiab]) OR "unplanned admission*"[tiab] OR "nonelective admission*"[tiab] OR "non-elective admission*"[tiab] OR "unexpected admission*"[tiab] OR "unscheduled admission*"[tiab] OR "nonplanned admission*"[tiab] OR "non-planned admission*"[tiab] OR "unanticipated admission*"[tiab] OR "emergency admission*"[tiab] OR "preventable admission*"[tiab] OR "unplanned visit*"[tiab] OR "nonelective visit*"[tiab] OR "non-elective visit*"[tiab] OR "unexpected visit*"[tiab] OR "unscheduled visit*"[tiab] OR "nonplanned visit*"[tiab] OR "non-planned visit*"[tiab] OR "unanticipated visit*"[tiab] OR "emergency visit*"[tiab] OR "preventable visit*"[tiab] OR "unplanned readmission*"[tiab] OR "nonelective readmission*"[tiab] OR "non-elective readmission*"[tiab] OR "unexpected readmission*"[tiab] OR "unscheduled readmission*"[tiab] OR "nonplanned readmission*"[tiab] OR "non-planned readmission*"[tiab] OR "unanticipated readmission*"[tiab] OR "emergency readmission*"[tiab] OR "preventable readmission*"[tiab] OR "unplanned re-admission*"[tiab] OR "nonelective re-admission*"[tiab] OR "non-elective re-admission*"[tiab] OR "unexpected re-admission*"[tiab] OR "unscheduled re-admission*"[tiab] OR "nonplanned re-admission*"[tiab] OR "non-planned re-admission*"[tiab] OR "unanticipated re-admission*"[tiab] OR "emergency re-admission*"[tiab] OR "preventable re-admission*"[tiab] OR "unplanned hospitali*"[tiab] OR "nonelective hospitali*"[tiab] OR "non-elective hospitali*"[tiab] OR "unexpected hospitali*"[tiab] OR "unscheduled hospitali*"[tiab] OR "nonplanned hospitali*"[tiab] OR "non-planned hospitali*"[tiab] OR "unanticipated hospitali*"[tiab] OR "emergency hospitali*"[tiab] OR "preventable hospitali*"[tiab] OR ("repeated"[tiab] AND "admission*"[tiab])	131,887

Embase.com Session Results (07 Jan 2021)

Search	Query	Items found
#6	**#5 AND [2013-3000]/py**	6,542
#5	**#4 NOT ('conference abstract'/it OR 'conference review'/it)**	10,193
#4	**#1 AND #2 AND #3**	16,645
#3	**(validat*:ab,ti,kw OR predict*:ti OR rule*:ab,ti,kw) OR (predict*:ab,ti,kw AND (outcome*:ab,ti,kw OR risk*:ab,ti,kw OR model*:ab,ti,kw)) OR ((history:ab,ti,kw OR variable*:ab,ti,kw OR criteria:ab,ti,kw OR scor*:ab,ti,kw OR characteristic*:ab,ti,kw OR finding*:ab,ti,kw OR factor*:ab,ti,kw) AND (predict*:ab,ti,kw OR model*:ab,ti,kw OR decision*:ab,ti,kw OR identif*:ab,ti,kw OR prognos*:ab,ti,kw)) OR (decision*:ab,ti,kw AND (model*:ab,ti,kw OR clinical*:ab,ti,kw OR 'statistical model'/exp)) OR (prognostic:ab,ti,kw AND (history:ab,ti,kw OR variable*:ab,ti,kw OR criteria:ab,ti,kw OR scor*:ab,ti,kw OR characteristic*:ab,ti,kw OR finding*:ab,ti,kw OR factor*:ab,ti,kw OR model*:ab,ti,kw))**	5,846,177
#2	**'home care'/exp OR 'community health nursing'/exp OR 'independent living'/exp OR 'very elderly'/exp/mj OR 'frail elderly'/exp/mj OR 'frailty'/exp/mj OR 'community dwell*':ab,ti,kw OR 'home dwell*':ab,ti,kw OR 'living at home':ab,ti,kw OR 'aging in place':ab,ti,kw OR 'ageing in place':ab,ti,kw OR ('independent*':ab,ti,kw AND ('living':ab,ti,kw OR 'housing':ab,ti,kw)) OR 'home care*':ab,ti,kw OR 'district nurs*':ab,ti,kw OR 'visiting nurs*':ab,ti,kw OR 'health visitor*':ab,ti,kw OR 'community health nurs*':ab,ti,kw OR 'elderly':ab,ti,kw OR 'eldest':ab,ti,kw OR 'late-life*':ab,ti,kw OR 'frail':ab,ti,kw OR 'frailty':ab,ti,kw OR 'old age*':ab,ti,kw OR 'oldest old*':ab,ti,kw OR 'senior*':ab,ti,kw OR 'senium':ab,ti,kw OR 'very old*':ab,ti,kw OR 'septuagenarian*':ab,ti,kw OR 'octagenarian*':ab,ti,kw OR 'octogenarian*':ab,ti,kw OR 'nonagenarian*':ab,ti,kw OR 'centarian*':ab,ti,kw OR 'centenarian*':ab,ti,kw OR 'supercentenarian*':ab,ti,kw OR 'older people':ab,ti,kw OR 'older subject*':ab,ti,kw OR 'older patient*':ab,ti,kw OR 'older age*':ab,ti,kw OR 'older adult*':ab,ti,kw OR 'older man':ab,ti,kw OR 'older men':ab,ti,kw OR 'older male*':ab,ti,kw OR 'older woman':ab,ti,kw OR 'older women':ab,ti,kw OR 'older female*':ab,ti,kw OR 'older population*':ab,ti,kw OR 'older person*':ab,ti,kw OR 'older individual*':ab,ti,kw OR 'aged**	881,656

Search	Query	Items found
	**person*':ab,ti,kw**	
#1	**('hospitalization'/exp/mj AND 'statistics and numerical data'/exp) OR ('hospital emergency service'/exp/mj AND 'statistics and numerical data'/exp) OR 'hospital admission'/exp/mj OR 'hospital readmission'/exp OR 'hospital admission*':ab,ti,kw OR 'hospital readmission*':ab,ti,kw OR 'hospital re-admission*':ab,ti,kw OR 'hospital entry*':ab,ti,kw OR 'hospital re-ent*':ab,ti,kw OR (hospitali*:ab,ti,kw AND predict*:ab,ti,kw) OR 'unplanned admission*':ab,ti,kw OR 'nonelective admission*':ab,ti,kw OR 'non-elective admission*':ab,ti,kw OR 'unexpected admission*':ab,ti,kw OR 'unscheduled admission*':ab,ti,kw OR 'nonplanned admission*':ab,ti,kw OR 'non-planned admission*':ab,ti,kw OR 'unanticipated admission*':ab,ti,kw OR 'emergency admission*':ab,ti,kw OR 'preventable admission*':ab,ti,kw OR 'unplanned visit*':ab,ti,kw OR 'nonelective visit*':ab,ti,kw OR 'non-elective visit*':ab,ti,kw OR 'unexpected visit*':ab,ti,kw OR 'unscheduled visit*':ab,ti,kw OR 'nonplanned visit*':ab,ti,kw OR 'non-planned visit*':ab,ti,kw OR 'unanticipated visit*':ab,ti,kw OR 'emergency visit*':ab,ti,kw OR 'preventable visit*':ab,ti,kw OR 'unplanned readmission*':ab,ti,kw OR 'nonelective readmission*':ab,ti,kw OR 'non-elective readmission*':ab,ti,kw OR 'unexpected readmission*':ab,ti,kw OR 'unscheduled readmission*':ab,ti,kw OR 'nonplanned readmission*':ab,ti,kw OR 'non-planned readmission*':ab,ti,kw OR 'unanticipated readmission*':ab,ti,kw OR 'emergency readmission*':ab,ti,kw OR 'preventable readmission*':ab,ti,kw OR 'unplanned re-admission*':ab,ti,kw OR 'nonelective re-admission*':ab,ti,kw OR 'non-elective re-admission*':ab,ti,kw OR 'unexpected re-admission*':ab,ti,kw OR 'unscheduled re-admission*':ab,ti,kw OR 'nonplanned re-admission*':ab,ti,kw OR 'non-planned re-admission*':ab,ti,kw OR 'unanticipated re-admission*':ab,ti,kw OR 'emergency re-admission*':ab,ti,kw OR 'preventable re-admission*':ab,ti,kw OR 'unplanned hospitali*':ab,ti,kw OR 'nonelective hospitali*':ab,ti,kw OR 'non-elective hospitali*':ab,ti,kw OR 'unexpected hospitali*':ab,ti,kw OR 'unscheduled hospitali*':ab,ti,kw OR 'nonplanned hospitali*':ab,ti,kw OR 'non-planned hospitali*':ab,ti,kw OR 'unanticipated hospitali*':ab,ti,kw OR 'emergency hospitali*':ab,ti,kw OR 'preventable hospitali*':ab,ti,kw OR ('repeated':ab,ti,kw AND 'admission*':ab,ti,kw)**	216,706

CINAHL (Ebsco) Session Results (07 Jan 2021)

Search	Query	Items found
S5	**S4 AND Limiters - Published Date: 20130101-20211231**	3,776
S4	**S1 AND S2 AND S3**	5,756
S3	TI (validat* OR predict* OR rule*) OR (predict* AND (outcome* OR risk* OR model*)) OR ((history OR variable* OR criteria OR scor* OR characteristic* OR finding* OR factor*) AND (predict* OR model* OR decision* OR identif* OR prognos*)) OR (decision* AND (model* OR clinical*)) OR (prognostic AND (history OR variable* OR criteria OR scor* OR characteristic* OR finding* OR factor* OR model*)) OR AB (validat* OR rule*) OR (predict* AND (outcome* OR risk* OR model*)) OR ((history OR variable* OR criteria OR scor* OR characteristic* OR finding* OR factor*) AND (predict* OR model* OR decision* OR identif* OR prognos*)) OR (decision* AND (model* OR clinical*)) OR (prognostic AND (history OR variable* OR criteria OR scor* OR characteristic* OR finding* OR factor* OR model*)) OR SU (validat* OR rule*) OR (predict* AND (outcome* OR risk* OR model*)) OR ((history OR variable* OR criteria OR scor* OR characteristic* OR finding* OR factor*) AND (predict* OR model* OR decision* OR identif* OR prognos*)) OR (decision* AND (model* OR clinical*)) OR (prognostic AND (history OR variable* OR criteria OR scor* OR characteristic* OR finding* OR factor* OR model*))	1,373,721
S2	(MH "Home Health Care") OR (MH "Home Nursing, Professional") OR (MH "Community Health Nursing+") OR (MH "Community Living+") OR (MM "Aged, 80 and Over+") OR (MM "Frail Elderly") OR (MM "Aged, Hospitalized") OR (MM "Frailty Syndrome") OR TI ("community dwell*" OR "home dwell*" OR "living at home" OR "aging in place" OR "ageing in place" OR ("independent*" AND ("living" OR "housing")) OR "home care*" OR "district nurs*" OR "visiting nurs*" OR "health visitor*" OR "community health nurs*" OR "elderly" OR "eldest" OR "late-life*" OR "frail" OR "frailty" OR "old age*" OR "oldest old*" OR "senior*" OR "senium" OR "very	364,615

Search	Query	Items found
	old*" OR "septuagenarian*" OR "octagenarian*" OR "octogenarian*" OR "nonagenarian*" OR "centarian*" OR "centenarian*" OR "supercentenarian*" OR "older people" OR "older subject*" OR "older patient*" OR "older age*" OR "older adult*" OR "older man" OR "older men" OR "older male*" OR "older woman" OR "older women" OR "older female*" OR "older population*" OR "older person*" OR "older individual*" OR "aged person*") OR AB ("community dwell*" OR "home dwell*" OR "living at home" OR "aging in place" OR "ageing in place" OR ("independent*" AND ("living" OR "housing")) OR "home care*" OR "district nurs*" OR "visiting nurs*" OR "health visitor*" OR "community health nurs*" OR "elderly" OR "eldest" OR "late-life*" OR "frail" OR "frailty" OR "old age*" OR "oldest old*" OR "senior*" OR "senium" OR "very old*" OR "septuagenarian*" OR "octagenarian*" OR "octogenarian*" OR "nonagenarian*" OR "centarian*" OR "centenarian*" OR "supercentenarian*" OR "older people" OR "older subject*" OR "older patient*" OR "older age*" OR "older adult*" OR "older man" OR "older men" OR "older male*" OR "older woman" OR "older women" OR "older female*" OR "older population*" OR "older person*" OR "older individual*" OR "aged person*") OR SU ("community dwell*" OR "home dwell*" OR "living at home" OR "aging in place" OR "ageing in place" OR ("independent*" AND ("living" OR "housing")) OR "home care*" OR "district nurs*" OR "visiting nurs*" OR "health visitor*" OR "community health nurs*" OR "elderly" OR "eldest" OR "late-life*" OR "frail" OR "frailty" OR "old age*" OR "oldest old*" OR "senior*" OR "senium" OR "very old*" OR "septuagenarian*" OR "octagenarian*" OR "octogenarian*" OR "nonagenarian*" OR "centarian*" OR "centenarian*" OR "supercentenarian*" OR "older people" OR "older subject*" OR "older patient*" OR "older age*" OR "older adult*" OR "older man" OR "older men" OR "older male*" OR "older woman" OR "older women" OR "older female*" OR "older population*" OR "older person*" OR "older individual*" OR "aged person*")	
S1	(MM "Hospitalization") OR (MM "Emergency Service+") OR (MM "Patient Admission") OR (MH "Readmission") OR TI ("hospital readmission*" OR "hospital re-admission*" OR "hospital entry*" OR "hospital re-ent*" OR (hospitali* AND predict*) OR "unplanned admission*" OR "nonelective admission*" OR "non-elective admission*" OR "unexpected admission*" OR "unscheduled admission*" OR "nonplanned admission*" OR "non-planned admission*" OR "unanticipated admission*" OR "emergency admission*" OR "preventable admission*" OR	82,451

Search	Query	Items found
	"unplanned visit*" OR "nonelective visit*" OR "non-elective visit*" OR "unexpected visit*" OR "unscheduled visit*" OR "nonplanned visit*" OR "non-planned visit*" OR "unanticipated visit*" OR "emergency visit*" OR "preventable visit*" OR "unplanned readmission*" OR "nonelective readmission*" OR "non-elective readmission*" OR "unexpected readmission*" OR "unscheduled readmission*" OR "nonplanned readmission*" OR "non-planned readmission*" OR "unanticipated readmission*" OR "emergency readmission*" OR "preventable readmission*" OR "unplanned re-admission*" OR "nonelective re-admission*" OR "non-elective re-admission*" OR "unexpected re-admission*" OR "unscheduled re-admission*" OR "nonplanned re-admission*" OR "non-planned re-admission*" OR "unanticipated re-admission*" OR "emergency re-admission*" OR "preventable re-admission*" OR "unplanned hospitali*" OR "nonelective hospitali*" OR "non-elective hospitali*" OR "unexpected hospitali*" OR "unscheduled hospitali*" OR "nonplanned hospitali*" OR "non-planned hospitali*" OR "unanticipated hospitali*" OR "emergency hospitali*" OR "preventable hospitali*" OR ("repeated" AND "admission*")) OR AB ("hospital readmission*" OR "hospital re-admission*" OR "hospital entry*" OR "hospital re-ent*" OR (hospitali* AND predict*) OR "unplanned admission*" OR "nonelective admission*" OR "non-elective admission*" OR "unexpected admission*" OR "unscheduled admission*" OR "nonplanned admission*" OR "non-planned admission*" OR "unanticipated admission*" OR "emergency admission*" OR "preventable admission*" OR "unplanned visit*" OR "nonelective visit*" OR "non-elective visit*" OR "unexpected visit*" OR "unscheduled visit*" OR "nonplanned visit*" OR "non-planned visit*" OR "unanticipated visit*" OR "emergency visit*" OR "preventable visit*" OR "unplanned readmission*" OR "nonelective readmission*" OR "non-elective readmission*" OR "unexpected readmission*" OR "unscheduled readmission*" OR "nonplanned readmission*" OR "non-planned readmission*" OR "unanticipated readmission*" OR "emergency readmission*" OR "preventable readmission*" OR "unplanned re-admission*" OR "nonelective re-admission*" OR "non-elective re-admission*" OR "unexpected re-admission*" OR "unscheduled re-admission*" OR "nonplanned re-admission*" OR "non-planned re-admission*" OR "unanticipated re-admission*" OR "emergency re-admission*" OR "preventable re-admission*" OR "unplanned hospitali*" OR "nonelective hospitali*" OR "non-elective hospitali*" OR "unexpected hospitali*" OR "unscheduled hospitali*" OR "nonplanned hospitali*" OR "non-planned hospitali*" OR "unanticipated hospitali*" OR "emergency hospitali*" OR "preventable hospitali*" OR ("repeated" AND	

Search	Query	Items found
	**"admission*")) OR SU ("hospital readmission*" OR "hospital re-admission*" OR "hospital entry*" OR "hospital re-ent*" OR (hospitali* AND predict*) OR "unplanned admission*" OR "nonelective admission*" OR "non-elective admission*" OR "unexpected admission*" OR "unscheduled admission*" OR "nonplanned admission*" OR "non-planned admission*" OR "unanticipated admission*" OR "emergency admission*" OR "preventable admission*" OR "unplanned visit*" OR "nonelective visit*" OR "non-elective visit*" OR "unexpected visit*" OR "unscheduled visit*" OR "nonplanned visit*" OR "non-planned visit*" OR "unanticipated visit*" OR "emergency visit*" OR "preventable visit*" OR "unplanned readmission*" OR "nonelective readmission*" OR "non-elective readmission*" OR "unexpected readmission*" OR "unscheduled readmission*" OR "nonplanned readmission*" OR "non-planned readmission*" OR "unanticipated readmission*" OR "emergency readmission*" OR "preventable readmission*" OR "unplanned re-admission*" OR "nonelective re-admission*" OR "non-elective re-admission*" OR "unexpected re-admission*" OR "unscheduled re-admission*" OR "nonplanned re-admission*" OR "non-planned re-admission*" OR "unanticipated re-admission*" OR "emergency re-admission*" OR "preventable re-admission*" OR "unplanned hospitali*" OR "nonelective hospitali*" OR "non-elective hospitali*" OR "unexpected hospitali*" OR "unscheduled hospitali*" OR "nonplanned hospitali*" OR "non-planned hospitali*" OR "unanticipated hospitali*" OR "emergency hospitali*" OR "preventable hospitali*" OR ("repeated" AND "admission*"))**	
