# Supplementary material for: Prediction models for the prediction of unplanned hospital admissions in community-dwelling older adults: A systematic review
Source: PLoS One. 2022 Sep 23;17(9):e0275116. doi: 10.1371/journal.pone.0275116 (PMC9506609; doi:10.1371/journal.pone.0275116)
Supplement: S2 File — (PDF) [file pone.0275116.s003.pdf]

# S2 File: CHARMS and PROBAST forms

### CHARMS Quality assessment and data extraction form

| **Details of study:** | | |
|---|---|---|
| **Source of data** | Source of data | |
| **Participants** | Participant eligibility and recruitment method | |
| | Participant description, incl geographic location | |
| | Study dates | |
| **Outcome(s) to be predicted** | Definition and method for measurement of outcome | |
| | Was the same outcome definition (and method for measurement) used in all patients? | |
| | Type of outcome | |
| | Was the outcome assessed without knowledge of the candidate predictors? | |
| | Were candidate predictors part of the outcome? | |
| | Time of outcome occurrence or summary of duration of follow-up | |
| **Candidate predictors** | Number and type of predictors | |
| | Definition and method for measurement of candidate predictors | |
| | Timing of predictor measurement | |
| | Were predictors assessed blinded for outcome, and for each other? | |
| | Handling of predictors in the modelling | |
| **Sample size** | Number of participants and number of outcomes | |
| | Events per variable | |
| **Missing data** | Number of participants with any missing value | |
| | Number of participants with missing data for each predictor | |
| | Handling of missing data | |
| **Model development** | Modelling method | |
| | Modelling assumptions satisfied | |
| | Method for selection of predictors for inclusion in multivariable modelling | |

| | | |
|---|---|---|
| | Method for selection of predictors during multivariable modelling and criteria used | |
| | Shrinkage of predictor weights or regression coefficients | |
| **Model performance** | Calibration and discrimination measures with confidence intervals | |
| | Classification measures and whether a-priori cut points were used | |
| **Model evaluation** | Method used for testing model performance: internal/external validation | |
| | In case of poor validation, whether the model was adjusted/updated | |
| **Results** | Final and other multivariable models presented, including predictor weights or regression coefficients, intercept, baseline survival, model performance measures | |
| | Any alternative presentation of the final prediction models | |
| | Comparison of the distribution of predictors for development and validation datasets | |
| **Interpretation and discussion** | Interpretation of presented models | |
| | Comparison with other studies, discussion of generalizability, strengths and limitations | |

## PROBAST Risk of bias and applicability assessment form

| | |
|---|---|
| **First author** | |
| **Domain 1: Participants** | |
| Were all appropriate data sources used?<br>Were all inclusions and exclusions of participants appropriate?<br>*Risk of bias:*<br>*Applicability:* | |
| **Domain 2: Predictors** | |
| Were predictors defined and assessed in a similar way for all participants?<br>Were predictor assessments made without knowledge of outcome data?<br>Are all predictors available at the time of the model is intended to be used?<br>*Risk of bias:*<br>*Applicability:* | |
| **Domain 3: Outcome** | |
| Was the outcome determined appropriately?<br>Was a pre-specified or standard outcome definition used?<br>Were predictors excluded from the outcome definition?<br>Was the outcome defined and determined in a similar way for all participants?<br>Was the outcome determined without knowledge of predictor information?<br>Was the time interval between predictor assessment and outcome determination appropriate?<br>*Risk of bias:*<br>*Applicability:* | |
| **Domain 4: Analysis** | |
| Were there a reasonable number of participants with the outcome?<br>Were continuous and categorical predictors handled appropriate?<br>Were all enrolled participants included in the analysis?<br>Were all enrolled participants with missing data handled appropriately?<br>Was selection of predictors based on univariable analyses avoided?<br>Were complexities in the data accounted for appropriately?<br>Were relevant model performance measures evaluated appropriately?<br>Were model overfitting and optimism in model performance accounted for?<br>Do predictors and their assigned weights in the final model correspond to the results from the reported multivariable analysis?<br>*Risk of bias:*<br>*Applicability:* | |
| **OVERALL:** | |
| Risk of bias:<br>Concern for applicability: | |
