# Supplementary material for: Prediction models for the prediction of unplanned hospital admissions in community-dwelling older adults: A systematic review
Source: PLoS One. 2022 Sep 23;17(9):e0275116. doi: 10.1371/journal.pone.0275116 (PMC9506609; doi:10.1371/journal.pone.0275116)
Supplement: Attachment — Submitted filename: Peer review.docx [file pone.0275116.s004.docx]

First of all I would like to thank and congratulate the authors on their work. The topic of this systematic review is very interesting and important. The manuscript lacks however, structure and does not always read well. See my suggestions and questions below to improve this work.

**Background:**

Use of “risk” prediction modeling is a confusing and uncommon terminology. Recommend to use solely prediction model. This will probably improve the readability of the manuscript.

A very large proportion of the introduction is being used to describe prediction models, big data and machine learning in general. This does not read well, and does not add very much value to the topic of this systematic review. I would recommend to explain more about the “burden” of older patients at the emergency department. For example, how many times are patients admitted to and ED?; What are the reasons that they visit the ED? Are these reasons preventable? This will highlight the importance of this research. Additionally, I would also recommend to focus on the effects of ED admission and hospitalization on the elderly, such as the loss of functionality, risk of delirium during admission, psychological effects etc. Additionally, the authors described that with an effective primary care intervention, healthcare costs will decrease. I suggest to add details about how identification of these elderly can improve the work of physicians on how they can deliver more qualitative and effective healthcare.

**Method:**

My major concern is the following: in the introduction and methods it is explained that previous reviews included studies focusing on ED admission and case-finding instruments. However, inclusion for this study was limited to studies from 2013 onwards, despite focusing on ED admission and unplanned hospitalization. The reason why the authors chose this specific inclusion year is confusing, as the previous performed reviews do not fully cover the research question of this systematic review. Could the authors explain, how and why this decision was made.

Methods section reads cloudy and could be more straightforward. For example: [1] *Since these systematic reviews identified the same risk prediction models, we decided to limit publication dates from August 2013 through January 2021, which has some overlap with the searches of these reviews. To give a complete overview, we will also include the studies found in the previous reviews. The references of the identified articles were searched for relevant publications.* I would suggest to rephrase to: [1] *To provide a complete overview of available prediction models our search was restricted to August 2013 through January 2021. The models described in the previous reviews were also included in this systematic review.*

[2] *After extraction of data, the Prediction model Risk of Bias Assessment Tool (PROBAST; see Appendix B) was used to assess risk of bias and applicability of the predictive models. Concern for applicability addresses whether the primary study matches the review question*. I would suggest to rephrase to: [2] *The Prediction model Risk of Bias Assessment Tool was used to assess risk of bias and applicability, of which the latter addresses whether the primary study matches the review question.*

I would suggest to change the structure of the methods section and shorten in. Combine sections search strategy, study selection and data extraction. Make a new subheading with model performance including de explanation about AUC and EPV and lastly discuss the PROBAST tool. The PROBAST tool is explained very extensively. I would recommend to remove details to supplements or just refer to original PROBAST article. Also describe that regression models and machine learning models will be described separately.

Inclusion criteria one, two and four seem obvious. However I think Inclusion criteria three and five need more explanation. In general a question to authors: why were only validated prediction models included in this study? With the PROBAST tool, the models are also scored on “validation”. I do not see why development studies are not included. In regards to inclusion criteria five: how do prediction models being used at the ED differ from the ones being used at a primary care facility?

**Results**

A question for authors: Did all of the included study focus on developing only 1 prediction model? Because in these kind of studies sometimes multiple models are developed/ validated and compared to each other. Is 22 studies equivalent to 22 unique prediction models?

**Textual comments**

Same as the methods. Results can be pointed out more straightforward. See examples below.

Line 162/168: *A flow diagram of the search strategy and selection process is presented in Figure 1,* can be removed. *Data extracted from the studies can be found in Table 1 and Table 2*. Suggest to change it to: *The literature searches yielded a total of 16,098 citations (Figure 1.).* Tables and figures do not a notification, referring is the standard.

Line 164. *Additionally, twenty-three articles were identified through other sources.* What are these other sources? This was not mentioned in the methods.

Line 165: *In addition to 10 studies included in the previously published systematic reviews, 12 new studies met all inclusion criteria, which makes a total of 22 unique risk prediction models.* Rephrase: *Full texts were retrieved for 170 studies of which 12 met all inclusion criteria. Additionally, a total of 10 studies were included from the previously published systematic reviews.*

I would suggest to refer to “prediction model” instead of “study” in the results sections. For example line 171: *Thirteen studies included participants aged ≥65 172 years[29, 33, 34, 36-40, 44, 46-49], the remaining studies used a higher age as inclusion criterion with.* Rephrase to: *Thirteen prediction models included……..*

When describing results, try to hold on to the structure in the methods. The EPV can be described in the “predictive accuracy” section.

Avoid using question marks in tables and figures. I would suggest to use NA or a color scheme, for example, high risk= red, low risk= green, unclear= purple.

**Discussion**

**Line 293:** *Twelve risk models were added to the existing evidence.* This does not read well. Suggest to remove this sentence.

Line 293-295: The recommendation of using nonmedical factors is never mentioned in the manuscript. This conclusion comes a bit out of the blue. I would recommend to make a more general conclusion, on the results that the authors did find (e.g. quality of models, performance of models etc.).

I do not understand why the difference between machine learning and logistic regression models is putted forward prominently in this article. In order to say whether one technique is superior to the other, you should validate both models in the identical population. In line 317 the authors state the machine learning techniques are not superior and in in line 328 the authors state that a fair comparison is not possible. Please be consequent in conclusions.

The discussion includes a lot of repetition of the results. Conclusions is solely based on the development of more prediction models. In the introduction the authors describe that the eventual goal is to develop a care management program to avoid these admission. The authors should highlight, how they could use these models to develop such a program. For example; are the variables in these model, standard measurements in a primary care facility?

If the conclusion is only developing more models, the authors should describe how to accomplish this. Which data source to use, which variables should be included, which modeling technique etc.
