# Supplementary material for: Prediction models for the prediction of unplanned hospital admissions in community-dwelling older adults: A systematic review
Source: PLoS One. 2022 Sep 23;17(9):e0275116. doi: 10.1371/journal.pone.0275116 (PMC9506609; doi:10.1371/journal.pone.0275116)
Supplement: Attachment — Submitted filename: Response to Reviewers.docx [file pone.0275116.s006.docx]

Reviewer #1: (No Response)

Reviewer #3: I would like to thank the authors for the revised manuscript. The manuscript has significantly improved and is well structured. The introduction contains all relevant information and emphasizes the relevance of this manuscript. However, I still have minor (profoundly textual comments) that can further improve this paper.

Line 193-196
Very long and hard to follow sentence. Suggest to rephrase.

- We assume the reviewer refers to the following sentence: “For calculations of the median predictive performance we only included the AUC for the outcome all-cause admission, except for the calculation of the median predictive performance per outcome (i.e. preventable and all-cause hospitalization), then AUCs for both outcomes were included.”

We agree this sentence causes confusion and have rephrased it:

*“The models developed for a specific type of hospitalization (i.e. preventable hospitalization or fall with hospitalization) (n=3), tended to perform better than the models for all-cause hospitalization (n=17), with a median AUC of 0.78 (range 0.74-0.78) versus 0.69 (range 0.61 - 0.76), respectively.* The two models that assessed AUCs for both outcomes (i.e. Tarekegn et al. and Wu et al.[37, 39]) were included in calculations of both medians with its corresponding AUC and were thus counted twice.”

Line 216
“additionally one study assessed fall with hospitalizations as outcome”.
As mentioned in the exclusion criteria, models developed for specific disease groups were excluded. I would suggest to remove this sentence in order the avoid confusion as the model also looked at ED visit and hospital admission.

- Thank you for your comment. We have removed any reference to the fall-related hospital admissions outcome of this model from the rest of the manuscript, as it is indeed an exclusion criterion mentioned in the methods section.

Line 276-277
Replace “and” by “an”

- Thank you for noticing the typo.

line 281-284
suggest to rephrase sentence.

- We have rephrased the sentence accordingly:

“Concern for applicability was high in three studies, because the study population or study outcome did not fully match the review question: one study only included older adults with a sensory impairment[29], one study excluded older adults with a hospital admission <6 months prior to the index date[45], and one study evaluated preventable hospital admissions as only outcome[46].”

Line 338-339
Instead of “narrowing” I would suggest the term “focusing”.

- Thank you, we have followed your suggestion.

Discussion
Line 343-344
Suggest to remove the result of AUC>0.8 for fall related hospital admission as suggested earlier. This model performance is namely for fall related hospitalizations.

- We have removed this sentence in the discussion.

The start of your discussion includes a lot of comparison with Wallace et al. I would suggest to narrow this part and only highlight the most important difference with an explanation. For example, in your results and discussion you mention that the predictive accuracy of the current models has significantly improved compared to the models in Wallace et al. Is there any explanation to this?

- Thank you for your suggestion. We have narrowed this paragraph and included a possible explanation to the increased predictive accuracy in the newer models.

“The new models had higher predictive accuracy than the older models. This might be explained by the fact that new models had larger sample sizes of the development cohort and also higher EPVs. Both are recommended by the TRIPOD, published in 2015 [16], to improve predictive accuracy and methodological quality. Moreover, the new models used administrative or clinical record data more often for the development of their model. Consistent with Wallace et al., we found that models developed using administrative or clinical record data had higher predictive accuracy than those developed using self-report data. Of the 10 new prediction models, eight used administrative data for development of their model.”

You also mention the further implications for future research. Should we indeed develop more models? And what about the nonmedical factors mentioned by Wallace at al. Could the authors maybe elaborate more on this topic in their discussion.

- Thank you. We have followed your suggestions and have rephrased the implications for future research paragraph. We put more emphasis on external validation and updating prediction models instead of developing new prediction models. Regarding updating of the models, we suggested the addition of nonmedical factors may contribute to an improved accuracy of the model. (Manuscript, pages 19-20, lines 320-344)
